# Nanocarrier-integrated multilayer films produced by 3D printing for improved skin adhesion and curcumin photostability

**DOI:** 10.3762/bjnano.17.30

**Published:** 2026-03-25

**Authors:** Thayse Viana de Oliveira, Ana Paula Farias Leão, Júlia Leão, Cesar Liberato Petzhold, Ruy Carlos Ruver Beck

**Affiliations:** 1 Programa de Pós-Graduação em Ciências Farmacêuticas, Faculdade de Farmácia, Universidade Federal do Rio Grande do Sul, Avenida Ipiranga, 2752, Porto Alegre, Rio Grande do Sul 90610-000, Brazil; 2 Laboratório de Nanocarreadores e Impressão 3D Em Tecnologia Farmacêutica (Nano3D), Faculdade de Farmácia, Universidade Federal do Rio Grande do Sul (UFRGS), Porto Alegre, Brazil; 3 Instituto de Química, Universidade Federal do Rio Grande do Sul (UFRGS), Av. Bento Gonçalves - Agronomia, Porto Alegre, RS 90650-001, Brazil

**Keywords:** additive manufacturing, bioadhesion, curcumin, nanocarriers, photostability

## Abstract

This proof-of-concept study evaluated whether semi-solid extrusion (SSE) 3D printing could be used to fabricate multilayer topical films that simultaneously enhance skin bioadhesion and photoprotection of curcumin, a highly photolabile anti-inflammatory and antioxidant compound. The development of topical films for cutaneous delivery faces several challenges, including the need for strong skin adhesion and the protection of photolabile actives from light exposure. We hypothesized that multilayered films designed for the cutaneous delivery of curcumin and produced by SSE could address these limitations. To overcome its poor solubility and enhance bioadhesion, curcumin was encapsulated in polymeric nanocapsules (C-NCs), yielding a mean particle size of 218 ± 5 nm, a polydispersity index of 0.10 ± 0.02, a zeta potential of −11 ± 4 mV, and 100% encapsulation efficiency. Films were fabricated containing either C-NCs (F_C-NC_) or unloaded curcumin (F_C_) and consisted of three layers, namely, a chitosan-based bottom layer, a middle layer of carboxymethylcellulose and alginate, and a carboxymethylcellulose top layer incorporating titanium dioxide (TiO_2_). The lower and intermediate layers contained C-NC or curcumin. The final films (15 × 15 × 1.5 mm) contained 282.20 ± 7.75 µg and 246.80 ± 6.70 µg of curcumin in F_C-NC_ and F_C_, respectively. Films containing the bottom chitosan layer exhibited the highest bioadhesion, while the presence of a TiO_2_ top layer effectively prevented UVC-induced photodegradation, supporting our hypothesis. Furthermore, the presence of C-NCs in F_C-NC_ films promoted higher bioadhesion. This proof-of-concept study demonstrates the feasibility of integrating nanocarriers with 3D printing technology to engineer multilayer polymeric films for cutaneous application, offering enhanced bioadhesion and photoprotection. This work demonstrates how additive manufacturing can be used to design hierarchically structured, nanocarrier-integrated systems with spatially resolved functionalities.

## Introduction

3D printing is an innovative technology that is poised to transform personalised medicine and healthcare manufacturing. For the healthcare industry, the use of 3D printing provides several key advantages, including the ability to adapt dosages according to a patient’s physiological characteristics and genetic background, as well as their individual response to drugs and active substances. This level of personalisation significantly minimises the likelihood of adverse drug reactions and enhances therapeutic efficacy. Furthermore, a wide range of pharmaceutical formulations, drug combinations, and release profiles can be developed in the form of either immediate-release or extended-release options [1–2]. Several 3D printing techniques can be applied to pharmaceutical development, including material jetting, fused deposition modelling, stereolithography, selective laser sintering, binder jetting, semi-solid extrusion (SSE), and direct powder extrusion [3–5].

SSE is a method in which a semi-solid material is placed into a syringe and continuously extruded layer by layer onto a smooth surface until the entire object is created. The semi-solid material itself consists of polymers, solvents, and excipients blended at an optimal ratio; the mixture should not be overly viscous to enable efficient extrusion from the syringe tip, but it must also not be too fluid in order to support the weight of each subsequent layer. After extrusion, a post-printing drying phase is required to eliminate the solvent. A significant advantage of this technique is that it can be performed at room temperature or even at lower temperatures, making it ideal for thermosensitive materials [6].

The SSE approach can be applied to produce various pharmaceutical dosage forms and can be tailored to the requirements of specific applications, such as the production of films intended to promote skin healing [7–8]. Although topical films can be an effective option for facilitating skin repair and wound healing, they must be carefully manufactured to maintain optimal moisture levels, which may vary depending on the wound type, such as venous ulcers or burns [9–12]. An ideal dressing should exhibit suitable bioadhesive properties, prevent bacterial growth, minimise pain, and control odour, while also being cost-effective and easy to replace. Current research therefore focuses on the development of specialised dressings using materials such as alginate and carboxymethylcellulose as alternatives to traditional gauze [13–14].

Bioactive molecules such as curcumin have shown promise for use in the promotion of skin repair and wound healing. Curcumin is a photosensitive polyphenol derived from *Curcuma longa* (turmeric) with well-documented biological activities [15–16], including properties that may be advantageous for skin-healing applications. For example, curcumin exhibits strong anti-inflammatory activity through inhibition of nuclear factor kappa B (NF-κB) signalling, as well as antioxidant activity through reactions with free radicals or reactive oxygen species (ROS) and inhibition of lipid peroxidation and DNA damage. These mechanisms contribute to reduced oxidative stress and enhanced wound-healing processes [17]. Consequently, curcumin is currently under investigation for incorporation into topical dosage forms such as emulsions, fibres, films, and hydrogels for wound-healing applications.

However, curcumin also exhibits low aqueous solubility, which limits its use in certain formulations [18]. Nanoparticles have the potential to overcome this limitation, as they enable the development of formulations with reduced particle sizes (typically between 100 and 500 nm) and tailored physicochemical properties [19–20]. In the pharmaceutical context, nanoparticles, including polymeric nanoparticles, have been extensively investigated as drug carriers. The use of nanosystems to enhance the bioactivity of curcumin was reported by Krausz et al., who demonstrated that a curcumin-loaded nanosystem inhibited the growth of methicillin-resistant *Staphylococcus aureus* (MRSA) and *Pseudomonas aeruginosa* in vitro in a dose-dependent manner, while also improving wound healing and inhibiting MRSA growth in vivo in a murine wound model [21]. More recently, Terzopoulou et al. developed collagen patches containing chitosan nanoparticles loaded with curcumin, which showed efficacy against psoriasis in in vitro assays [22]. Similarly, Olmos-Juste et al. employed 3D printing to produce alginate–cellulose nanofibre patches exhibiting adequate disintegration and in vitro release properties for the local administration of curcumin [23].

Although curcumin-loaded chitosan or alginate films have been previously reported, no studies have explored the deliberate spatial distribution of functional components enabled by multilayer 3D printing, nor the incorporation of a photoprotective top layer to shield curcumin from UV-induced degradation. Thus, the innovation of the present work lies not in the individual materials themselves, but in their integration into a rationally designed, layer-by-layer system produced through additive manufacturing.

At present, specific strategies to protect curcumin in topical films from UV-induced degradation have not been extensively investigated, and studies focusing on improving the skin-adhesion properties of curcumin-containing topical films remain limited. Therefore, in the present study, we designed 3D-printed topical films comprising three distinct layers, namely, (i) a bottom layer composed of a chitosan gel containing curcumin, (ii) an intermediate layer composed of carboxymethylcellulose, alginate gel, and curcumin, and (iii) a top layer composed of carboxymethylcellulose and titanium dioxide (TiO_2_). Curcumin was incorporated into the formulations either in its encapsulated form, loaded into polymeric nanocapsules, or non-encapsulated and dissolved in a hydroalcoholic solution. The preparation of films containing curcumin-loaded polymeric nanocapsules or non-encapsulated curcumin was based on previous studies demonstrating the ability of nanocarriers to improve the aqueous solubility and antioxidant activity of curcumin when formulated as a gel [24–26].

Consequently, the objective of the present study was to use SSE 3D printing to design and produce, for the first time, three-layer films composed of chitosan, carboxymethylcellulose, and alginate, containing either nanoencapsulated curcumin loaded into polymeric nanocapsules or non-encapsulated curcumin added from solution. The advantages of producing 3D-printed polymeric films compared with previously developed hydrogels for skin delivery include improved control over the applied curcumin dose, as well as the personalisation of the size and shape of the printed films for the treatment of specific skin areas, such as wounds.

In this proof-of-concept study, we hypothesised that SSE 3D printing could be strategically employed to construct multilayer topical films capable of addressing two key limitations of curcumin for cutaneous delivery, namely, (i) its pronounced photosensitivity, which necessitates a protective barrier and (ii) its limited affinity for the skin surface, which requires enhanced bioadhesion to maintain residence time. To test this hypothesis, we designed a trilayer architecture in which a chitosan-rich lower layer provides muco- and bioadhesive properties, while a TiO_2_-containing upper layer acts as a physical photoprotective barrier, and curcumin is incorporated either in a nanoencapsulated form or as a solubilised active. The scope of the present work did not include in vitro release or permeation studies, as the primary aim was to validate the structural and functional feasibility of a multilayer 3D-printed design; biopharmaceutical evaluations will be addressed in subsequent studies.

## Experimental

### Materials

Curcumin (purity level: 65%), poly(ε-caprolactone) (PCL), sorbitan monooleate, sodium alginate (≈200–300 kDa), and low-molecular-weight chitosan (50–190 kDa) were purchased from Sigma-Aldrich (São Paulo, Brazil). Sodium carboxymethylcellulose 4,000 cP (Na-CMC) was obtained from Levviale (São Paulo, Brazil). Titanium dioxide in the rutile form (Eusolex^®^ T-AVO) was obtained from Alpha Química. Acetone was obtained from Neon Comercial Ltda (São Paulo, Brazil), grape seed oil was purchased from Delaware Importadora Química (Porto Alegre, Brazil), and polysorbate 80 was purchased from Henrifarma (São Paulo, Brazil). HPLC-grade solvents, acetonitrile and methanol, were acquired from Merck Millipore (Porto Alegre, Brazil). Trifluoroacetic acid was obtained from Sigma-Aldrich (São Paulo, Brazil). All reagents and solvents were of pharmaceutical or HPLC grade and were used as received.

### Nanocapsule suspensions

#### Preparation

Nanocapsules were prepared using a preformed polymer interfacial deposition method that was previously described by our group, but with some modifications [27]. Curcumin-loaded polymeric nanocapsules (C-NC) were prepared using an organic phase composed of 0.010 g of curcumin, 0.1 g of PCL, 27 mL of acetone, 0.0390 g of sorbitan monooleate and 165 µL of grape seed oil, under magnetic stirring at approximately 400 rpm for 4 h at 40 °C. After complete solubilisation of all components, the organic phase was combined by pouring it into the aqueous phase under moderate stirring (the temperature and agitation speed parameters were the same as those of the organic phase). The aqueous phase was composed of 0.077 g of polysorbate 80 and 54 mL of ultrapure water. Acetone was removed under reduced pressure, and the resulting suspensions were concentrated to 10 mL. The experiment was carried out in a dark environment, protected from light. Blank formulations (B-NC) were prepared under the same conditions, without addition of curcumin in the organic phases. All formulations were prepared and characterised in triplicate.

#### Particle size, pH and zeta potential

The z-average particle size and the polydispersity index were measured by dynamic light scattering using a Zetasizer^®^ Nano ZS (ZEN 3600, Malvern Instruments, USA). For this analysis, 10 µL of the C-NC formulation was diluted in 5 mL of ultrapure water (previously filtered through a 0.45 µm hydrophilic membrane, Millipore^®^), corresponding to a 500-fold dilution, as shown in Equation 1. The zeta potential was determined by measuring electrophoretic mobility at 25 °C using the same instrument (Zetasizer^®^ Nano ZS, ZEN 3600, Malvern Instruments, USA). Prior to analysis, the samples were diluted 500-fold in an aqueous 10 mmol/L NaCl solution and filtered through a 0.45 µm membrane (Millipore^®^). The pH of the formulations was measured without prior dilution at 25 °C using a calibrated potentiometer (VB-10, Denver Instrument, USA).


[1] $$C_{2}=\frac{C_{1}\cdot V_{1}}{V_{2}},$$

where *C*_2_ is the concentration of the sample after dilution, *C*_1_ is the initial concentration of the nanocapsule suspension, *V*_1_ is the volume of the nanocapsule suspension that was used, and V_2_ is the final volume of the sample.

#### Curcumin content and encapsulation efficiency

The curcumin content was assayed by high-performance liquid chromatography with ultraviolet detection (HPLC-UV) [26]. The chromatographic system consisted of a Shimadzu LC-20A chromatograph (Tokyo, Japan), using a C18 reversed-phase column (250 × 4.60 mm, 5 µm, 100 Å; Zorbax Eclipse Plus, Agilent). The mobile phase was composed of acetonitrile and trifluoroacetic acid (0.1%) (60:40, v/v), and the pH was adjusted to 3.0 using triethanolamine. Chromatography was conducted at a flow rate of 1.0 mL/min, with an injection volume of 20 µL, and curcumin was detected at a wavelength of 427 nm. The method was linear over the concentration range of 2.5–50.0 µg/mL of curcumin (*y* = 126634*x* − 12894; *r* = 0.9994), with intra-day variability lower than 2% and limits of detection and quantification of 0.2 and 0.7 µg/mL, respectively. Method specificity was confirmed by comparing chromatograms of the samples with those obtained from the placebo formulation. The curcumin content was determined after extraction with acetonitrile for 20 min, followed by filtration through a 0.45 µm membrane (Millipore^®^) prior to HPLC analysis. All analytical procedures were performed under dark conditions to protect the samples from light.

Encapsulation efficiency was determined using an ultrafiltration–centrifugation technique (Amicon^®^ 10,000 MW cutoff, Millipore^®^, Billerica, USA). Non-encapsulated curcumin was separated from curcumin-loaded nanocapsules using an ultrafiltration device, followed by centrifugation at 2150*g* for 10 min. Encapsulation efficiency was calculated as the difference between the total curcumin content and the non-encapsulated curcumin content.

#### Production of the printing Inks

Three different polymeric hydrogels or hydrogel blends were produced, corresponding to the three distinct layers of the film. Table 1 presents the composition of the different hydrogels prepared and used as printing inks for the present study. Some hydrogels were prepared solely for comparative purposes and were used during the characterisation step. Chitosan was the polymer used to produce the hydrogel for the bottom layer of the film. This natural polymer was initially mixed with glycerine in a porcelain mortar, and either C-NC (curcumin-loaded polymeric nanocapsules) or C (curcumin) solubilised in a hydroalcoholic solution (1:1, v/v) was slowly added. After complete homogenisation, 3% (v/v) acetic acid was added to solubilise the chitosan. For the intermediate layer, hydrogels of sodium carboxymethylcellulose (Na-CMC) and sodium alginate were produced separately. Na-CMC was dispersed in either C-NC or C until complete gelation was achieved. The sodium alginate hydrogel was produced using the same procedure, but with the addition of calcium carbonate to induce gelation. The hydrogels were then combined and mixed in a porcelain mortar at a ratio of 3.5:1 (w/w) Na-CMC/alginate, with the addition of glycerine, and the resulting blend was used as the printing ink. For the top layer, Na-CMC was mixed with glycerine and solubilised in purified water in a porcelain mortar until gel formation was achieved. Titanium dioxide (TiO_2_) was then added, and the gel mixture was completely homogenised using a pestle.

**Table 1:** Composition of the printing inks (hydrogels) for the SSE printing process.^a^

	Alginate (%)	Na-CMC (%)	Chitosan (%)	TiO_2_ (%)	Glycerine (%)	C-NC (%)	C (%)	H_2_O (%)

HG-Chi_C-NC_	—	—	7	—	10	83	—	—
HG-CMC/Alg_C-NC_	5	6	—	—	10	79	—	—
HG-Chi_C_	—	—	7	—	10	—	83	—
HG-CMC/Alg_C_	5	6	—	—	10	—	79	—
HG-Chi	—	—	7	—	10	—	—	83
HG-CMC/Alg	5	6	—	—	10	—	—	79
HG-CMC/TiO_2_	—	6	—	1	10	—	—	83

^a^HG-Chi_C-NC_: chitosan hydrogel containing curcumin-loaded polymeric nanocapsules; HG-CMC/Alg_C-NC_: sodium carboxymethylcellulose and alginate hydrogel blend containing curcumin-loaded polymeric nanocapsules; HG-Chi_C_: chitosan hydrogel containing curcumin added as hydroalcoholic solution; HG-CMC/Alg_C_: sodium carboxymethylcellulose and alginate hydrogel blend containing curcumin added as hydroalcoholic solution; HG-Chi: chitosan hydrogel; HG-CMC/Alg: sodium carboxymethylcellulose and alginate hydrogel blend; HG-CMC/TiO_2_: sodium carboxymethylcellulose and alginate hydrogel blend containing titanium dioxide.

#### pH measurements

After dispersion of each hydrogel in water (10% w/v), the pH of the resulting printing inks was measured at 25 °C using a calibrated potentiometer (VB-10, Denver Instrument, USA).

#### Rheological analyses

Rheological properties of all hydrogels were evaluated using an ARES G2 rheometer (TA Instruments, New Castle, DE, USA) equipped with a parallel-plate geometry (25 mm diameter, 0.9 mm gap) at 20 °C. Viscoelastic properties, including the storage modulus (*G*′), loss modulus (*G*″), and loss tangent (tan δ), were measured using an oscillatory frequency sweep over an angular frequency range of 1–600 rad/s at a constant strain of 0.5%. Flow behaviour was assessed using a shear rate ramp from 0.1 to 1.0 s^−1^ and subsequently from 1.0 to 0.1 s^−1^, in order to determine the best-fit flow model for the hydrogels. An oscillatory amplitude sweep was performed over a strain range of 0.1–500% at a constant frequency of 1 Hz to determine the linear viscoelastic region and to estimate the yield stress. Thixotropic behaviour was evaluated using a three-interval thixotropy test (3ITT), conducted as follows: the first interval was performed at 1.0% strain for 100 s, the second interval at 100% strain for 100 s, and the third interval at 1.0% strain for 100 s. All measurements were performed in triplicate.

### Topical skin films

#### 3D printing

Three-layer films containing curcumin-loaded polymeric nanocapsules (F_C-NC_) or curcumin solubilised in a hydroalcoholic solution (F_C_) were 3D-printed by semi-solid extrusion using a BioEdTech printer (São Paulo, Brazil) and three different hydrogels as printing inks. HG-Chi was used as the printing ink to produce the bottom layer of the film. Subsequently, two intermediate layers were printed using HG-CMC/Alg on top of the bottom layer, followed by the deposition of two upper layers using HG-CMC/TiO_2_. Because the 3D printer was equipped with a single printing head, the syringe containing the printing ink had to be manually replaced between layers with the syringe containing the hydrogel corresponding to the layer to be printed; this procedure required less than 45 s. The film geometry was designed using PrusaSlicer software (Prusa Research, Prague, Czech Republic), with final dimensions of 15 mm (length) × 15 mm (width) × 1.5 mm (height). The printer recognised the generated model and, using a syringe fitted with a nozzle with a diameter of 0.41 mm, deposited the material layer by layer at room temperature. The printing parameters were set to a rectilinear infill pattern with 100% infill density and an extrusion speed of 6 mm/s. After printing, the films were dried at 25 °C for 48 h to allow for water evaporation.

#### Physical characterisation

The physical properties of the resulting 3D-printed films (*n* = 10) were measured. Namely, films were individually weighed on an analytical balance (Shimadzu, Tokyo, Japan), and their length, width and height were measured using a digital calliper (Digimess, São Paulo, Brazil).

#### Curcumin content

The curcumin content of the films (*n* = 3) was determined by transferring a single weighed unit to 10 mL of ultrapure water in a volumetric flask of 25 mL, followed by mixing under magnetic stirring for 1 h, until complete disintegration. After this period, the volume of the volumetric flask was adjusted to 25 mL with methanol and sonicated for 2 h. Then, a 0.5 mL aliquot was removed and transferred to a 5 mL volumetric flask and diluted with the mobile phase. These samples were filtered through a 0.45 µm membrane, and the level of curcumin was assayed by HPLC using the method described in section “Curcumin content and encapsulation efficiency“. The entire assay was conducted under protection from light.

#### Differential scanning calorimetry

Differential scanning calorimetry (DSC) was performed using a differential scanning calorimeter (Shimadzu DSC-60), at a heating rate of 10 °C/min, from room temperature to 300 °C. N_2_ gas was used at a flow rate of 50 mL/min. All film components (chitosan, CMC and alginate), as well as the resulting films (F_C-NC_ and F_C_) were analysed separately.

#### Skin adhesion measurements

Bioadhesion tests of the complete 3D-printed films (F_C-NC_ and F_C_, *n* = 3) were conducted using a texture analyser (TA.XT plus, Stable Micro Systems, Godalming, Waverley, UK). To evaluate the influence of chitosan, the bioadhesion of films containing curcumin or nanoencapsulated curcumin without a chitosan layer (F_C-NC w/o CH_ and F_C w/o CH_, *n* = 3) was also evaluated using porcine ear skin as model. The ears were donated by Ouro do Sul Cooperative, Harmonia, Rio Grande do Sul, Brazil. The ear skins were cut, removing the hair and adipose tissue. The skins were stored in aluminium foil at −20 °C until the day of the experiment, without using any media or further treatment. On the day of the experiments, skin tissue was maintained under room conditions for at least 30 min prior to the experiments. A volume (20 µL) of ultrapure water was added by pipette onto the tissue to ensure hydration, and excess water was removed with absorbent paper. Skin samples were fixed to the equipment probe with instantaneous adhesive, while films were fixed to the equipment’s platform with double-sided tape. The equipment promoted contact between the skin sample and the film, with a force of 290 mN for 3 min. The probe holding the skin sample was then withdrawn from the surface of the film by the platform at a constant speed of 0.10 mm/s until total displacement was achieved. The work required to detach the skin sample from the 3D-printed films was calculated based on the peak force and maximum displacement after complete detachment using software (Exponent, Stable Micro Systems, Godalming, Waverley, UK) [28].

#### Photodegradation behaviour

Photodegradation analyses were conducted in a mirrored chamber under an UVC lamp for 24 h, and all analyses were done in triplicate. UVC irradiation was selected as a stress condition to perform a forced degradation photostability challenge, in line with accelerated conditions commonly employed to quantify intrinsic photolability of highly photosensitive compounds such as curcumin. This approach provides an exaggerated but controlled environment to compare the relative protective effects of the TiO_2_-containing layer. It was not intended to mimic physiological solar exposure, and future assessments under UVA/UVB spectra are planned. Four groups (a–d) were evaluated as follows: (a) complete films containing nanoencapsulated curcumin (F_C-NC_), (b) complete films containing unloaded curcumin (F_C_), (c) films containing nanoencapsulated curcumin (F_C-NC_) without an upper TiO_2_-containing carboxymethylcellulose layer, and (d) films containing unloaded curcumin (F_C_) without an upper TiO_2_-containing carboxymethylcellulose layer. Films (*n* = 27) from each group were placed in the UVC chamber for 24 h. At 2, 4, 6, 8, 10, 18, and 24 h, three films were removed from the chamber and underwent the curcumin extraction process and HPLC assay using the methods described in section “Curcumin content and encapsulation efficiency“.

### Statistical analyses

Statistical analyses were performed using one-way analysis of variance (ANOVA) followed by a post hoc Tukey’s test for multiple comparisons of means. Differences were considered significant at *p* ≤ 0.05.

## Results

### Nanocapsule suspensions

Curcumin-loaded polymeric nanocapsules (C-NC) and blank nanocapsules without curcumin (B-NC) were produced (*n* = 3). Table 2 provides a summary of results from physicochemical characterization.

**Table 2:** Physicochemical properties of curcumin-loaded nanocapsules (C-NC) and corresponding blank nanocapsule (B-NC) formulations.

	C-NC	B-NC

z-average (nm)	218 ± 5	230 ± 8
polydispersity index	0.11 ± 0.02	0.10 ± 0.02
zeta potential (mV)	−11 ± 4	−10 ± 1
pH	6.4 ± 0.1	6.9 ± 0.1
encapsulation efficiency (%)	100 ± 0.00	—
curcumin content (mg/mL)	0.98 ± 0.03	—

### Printing ink

**Figure 1:**
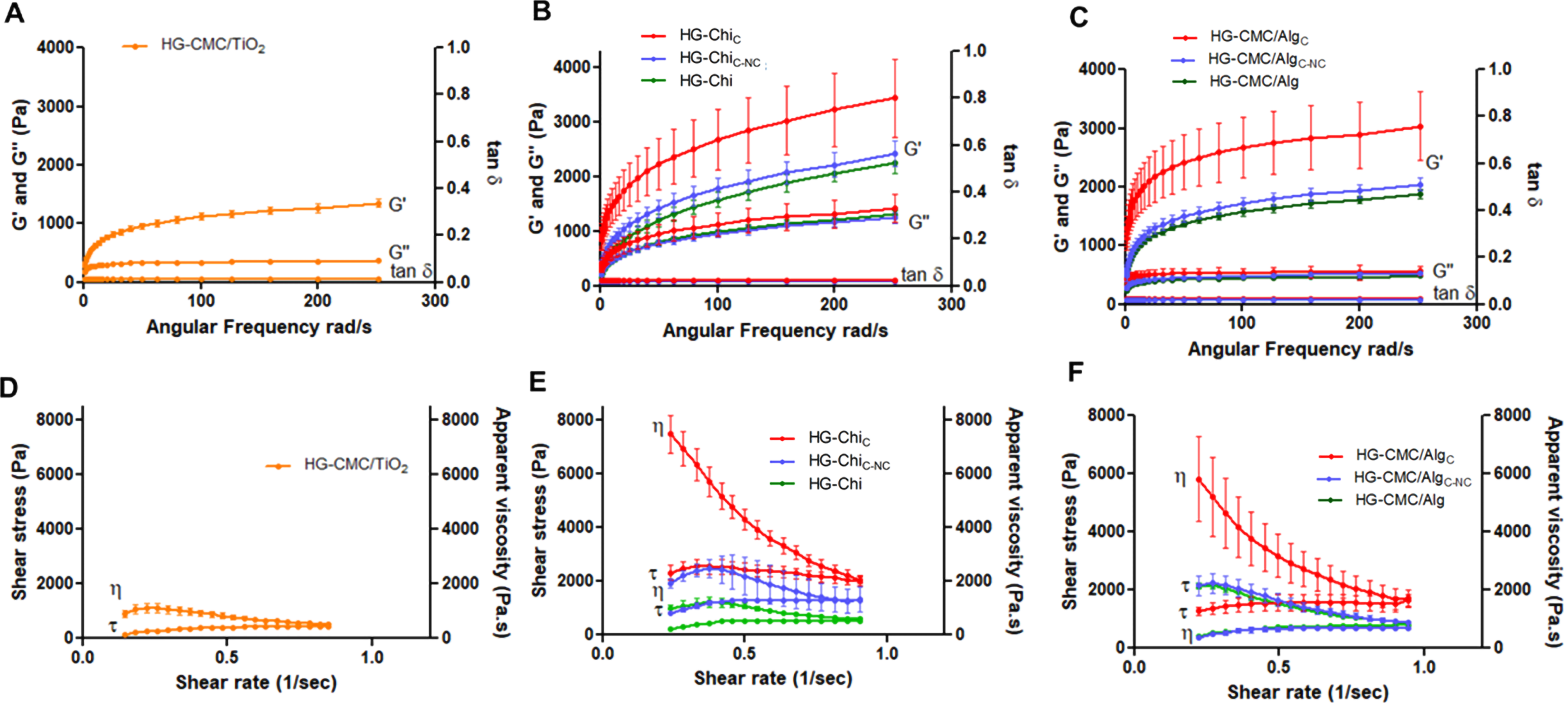
(A–C) Rheological behaviour with oscillation frequency data for storage (G′), loss modulus (G″), and tangent delta (tan δ) versus angular frequency and (D–F) flow curves presented as shear stress (τ) and apparent viscosity (η) versus shear rate of (A, D) the blend composed of sodium carboxymethylcellulose and alginate hydrogels containing titanium dioxide (HG-CMC/TiO_2_); (B, E) chitosan hydrogels containing unloaded curcumin (HG-Chi_C_), chitosan hydrogel containing curcumin-loaded polymeric nanocapsules (HG-Chi_C-nc_) and chitosan hydrogel (HG-Chi), and (C, F) blend of sodium carboxymethyl cellulose and alginate hydrogels containing curcumin in hydroalcoholic solution (HG-CMC/Alg_C_), blend of sodium carboxymethylcellulose and alginate hydrogels containing curcumin-loaded polymeric nanocapsules (HG-CMC/Alg_C-NC_), and sodium carboxymethylcellulose and alginate hydrogel (HG-CMC/Alg).

During the 3D printing process, the rheological behaviour of a printing ink is directly related to printability and the ability to form stable, structured layers, in order to avoid collapse after addition of subsequent layers. Because of this, the rheological properties of the hydrogels tested in the present study were investigated, and results are shown in Table 3. Based on a frequency oscillation test, as shown in Figure 1A–C, all samples had G′ values greater than G″ and, hence, a tan δ (ratio of G″/G′) below 1. Flow curves presented as shear stress (τ) and apparent viscosity (η) versus shear rate are shown in Figure 1D–F. Finally, Table 4 shows the thixotropic properties of the hydrogels, demonstrating recovery of the viscosity of the hydrogels after stress cessation.

**Table 3:** Yield stress presented as the crossover modulus and complex viscosity of the hydrogels at 1 Hz (frequency sweep test). Values are presented as the mean ± SD (*n* = 3).^a^

	Yield stress (Pa)	Complex viscosity (Pa·s)

HG-Chi_C-NC_	332.33 ± 18.18	136.19 ± 12.88
HG-CMC/Alg_C-NC_	391.34 ± 39.23	193.27 ± 18.77
HG-Chi_C_	539.89 ± 31.12	310.60 ± 22.07
HG-CMC/Alg_C_	758.43 ± 180.82	469.51 ± 73.63
HG-Chi	352.45 ± 27.94	102.26 ± 11.69
HG-CMC/Alg	366.90 ± 6.30	187.62 ± 1.53
HG-CMC/TiO_2_	216.26 ± 12.78	101.55 ± 8.97

^a^HG-Chi_C-NC_: chitosan hydrogel containing curcumin-loaded polymeric nanocapsules; HG-CMC/Alg_C-NC_: a blend of sodium carboxymethylcellulose and alginate hydrogel containing curcumin-loaded polymeric nanocapsules; HG-Chi_C_: chitosan hydrogel containing curcumin in a hydroalcoholic solution; HG-CMC/Alg_C_: a blend of sodium carboxymethylcellulose and alginate hydrogel containing curcumin in a hydroalcoholic solution; HG-Chi: chitosan hydrogel; HG-CMC/Alg: a blend of sodium carboxymethylcellulose and alginate hydrogel; HG-CMC/TiO_2_: a blend of sodium carboxymethylcellulose and alginate hydrogel containing titanium dioxide.

**Table 4:** Percentage of recovery after stress cessation to evaluate the thixotropic properties of the hydrogels (*n* = 3).^a^

	Recovery (%)

HG-Chi_c-nc_	94.44 ± 2.30
HG-CMC/Alg_c-nc_	101.05 ± 1.56
HG-Chi_C_	97.14 ± 0.62
HG-CMC/Alg_C_	93.95 ± 1.70
HG-Chi	99.36 ± 0.21
HG-CMC/Alg	98.99 ± 0.27
HG-CMC/TiO_2_	98.92 ± 0.12

^a^HG-Chi_C-nc_: chitosan hydrogel containing curcumin-loaded polymeric nanocapsules; HG-CMC/Alg_C-nc_: blend of sodium carboxymethylcellulose and alginate hydrogels containing curcumin-loaded polymeric nanocapsules; HG-Chi_C_: chitosan hydrogel containing curcumin in a hydroalcoholic solution; HG-CMC/Alg_C_: blend of sodium carboxymethylcellulose and alginate hydrogels containing curcumin in a hydroalcoholic solution; HG-Chi: chitosan hydrogel; HG-CMC/Alg: blend of sodium carboxymethylcellulose and alginate hydrogels; HG-CMC/TiO_2_: blend of sodium carboxymethylcellulose and alginate hydrogels containing titanium dioxide.

### 3D Printing of three-layer films

**Figure 2:**
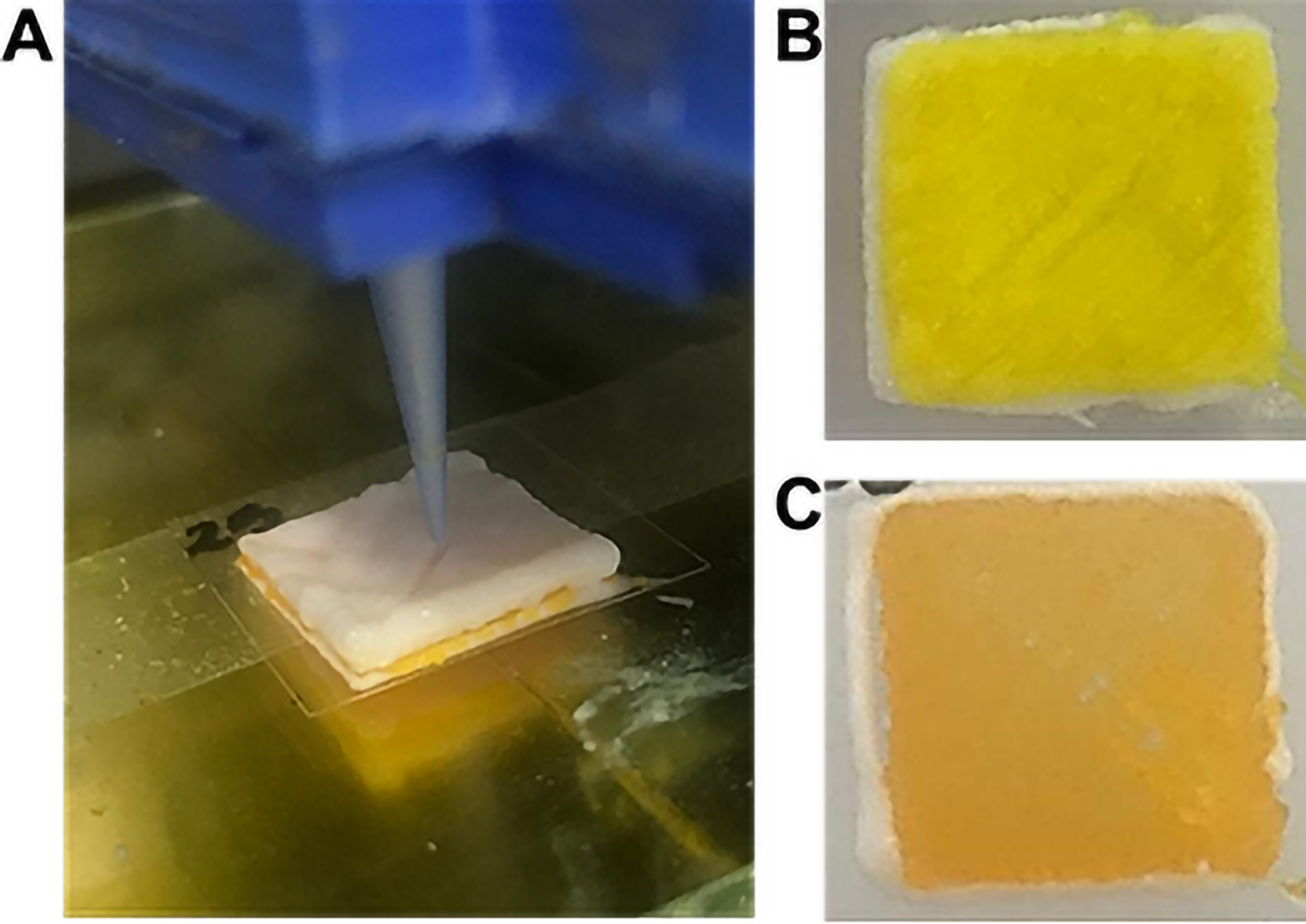
Three-layer 3D-printed film: (A) top view of the 3D-printed film; (B) bottom view of the 3D-printed film containing curcumin-loaded nanocapsules (F_C-NC_); (C) bottom view of the 3D-printed film containing unloaded curcumin (F_C_).

Two topical films composed of three distinct hydrogel layers were 3D-printed; examples are shown in Figure 2. Although the printer was equipped with a single extrusion head, syringe replacement was performed rapidly (typically faster than 45 s), and the printed construct was maintained under controlled ambient humidity to prevent premature surface drying. The viscoelastic properties of the hydrogels ensured that the previously deposited layer remained tacky and capable of bonding with the subsequent layer. Interlayer continuity was qualitatively verified by cross-sectional inspection, which confirmed the absence of visible delamination. The 3D-printed films exhibited a white top layer, composed of Na-CMC and TiO_2_ (Figure 2A), and bottom and intermediate layers that were visually similar, displaying yellow coloration due to the presence of either curcumin-loaded nanocapsules (F_C-NC_) (Figure 2B) or unloaded curcumin (F_C_) (Figure 2C).

**Figure 3:**
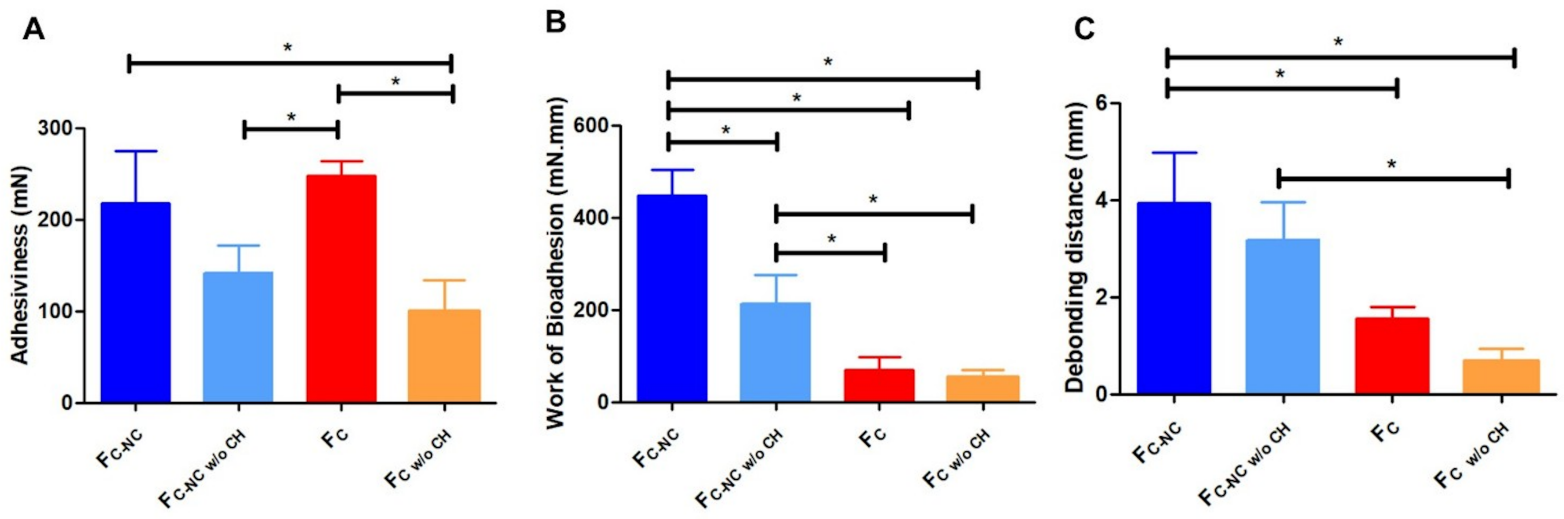
Bioadhesion measurements: (A) adhesiveness (mN), (B) bioadhesion work (mN·mm), and (C) debonding distance (mm) of the 3D-printed films with (F_C-NC_ and F_C_) and without chitosan (F_C-NC w/o CH_ and F_C w/o CH_). *Statistically significant (*p* ≤ 0.05) (ANOVA).

Figure 3 presents the results of the skin adhesion tests performed with the four film formulations, namely, films containing curcumin-loaded nanocapsules (F_C-NC_) or unloaded curcumin (F_C_), and films containing curcumin-loaded nanocapsules without a chitosan layer (F_C-NC w/o CH_) or unloaded curcumin without a chitosan layer (F_C w/o CH_). When measurable adhesiveness was observed (Figure 3A), the corresponding work of bioadhesion (Figure 3B) and the distance required to completely separate the skin sample from the film (Figure 3C) were determined.

**Figure 4:**
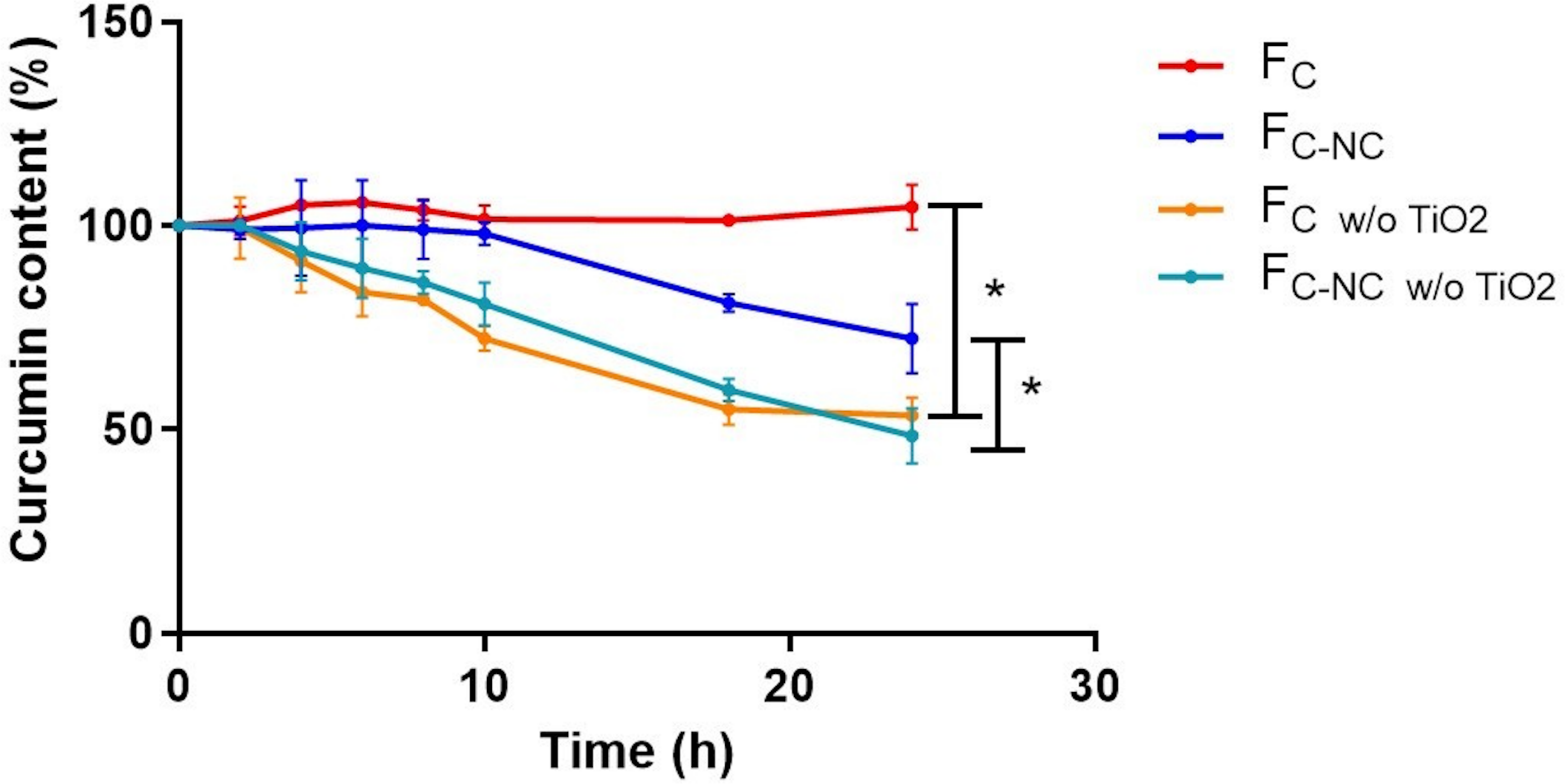
Photodegradation profile of curcumin during UV exposure of the following 3D-printed films: with addition of TiO_2_ in the 3D-printed films (F_C-NC_: films containing curcumin-loaded nanocapsules, F_C_: films containing unloaded curcumin) and without TiO_2_ (F_C-NC w/o TiO2_: films containing curcumin-loaded nanocapsules without TiO_2_, F_C w/o TiO2_: films containing unloaded curcumin without TiO_2_) *Statistically significant (*p* ≤ 0.05) (ANOVA).

Figure 4 provides a comparative analysis of curcumin photodegradation across four experimental groups, allowing for discrimination between the effects of nanoencapsulation and the TiO_2_-containing top layer. Films lacking the TiO_2_ layer (F_C-NC w/o TiO2_ and F_C w/o TiO2_) exhibited a progressive and statistically significant reduction in curcumin content under UVC exposure, confirming the intrinsic photolability of curcumin in the polymeric matrix. In contrast, complete multilayer films incorporating the TiO_2_-containing top layer maintained significantly higher curcumin levels over 24 h, demonstrating the effective photoprotective function of this external barrier.

Notably, while nanoencapsulation alone did not fully prevent photodegradation in the absence of TiO_2_, F_C-NC w/o TiO2_ showed slightly improved retention compared with F_C w/o TiO2_, suggesting that nanocarrier confinement may provide partial protection by restricting molecular mobility and reducing direct light exposure within the matrix. However, the most pronounced protective effect was clearly associated with the presence of the TiO_2_-containing layer, indicating that spatial separation of functions enabled by multilayer 3D printing is critical for achieving effective photostabilization.

## Discussion

Based on the observed physicochemical properties (Table 2), the formulations developed in the present study exhibited a low polydispersity index and a z-average particle size below 240 nm, indicating a narrow nanometric size distribution. All tested formulations presented a negative zeta potential. In addition to the low magnitude of the negative zeta potential, physical stability was further ensured by steric stabilisation, resulting from the presence of a non-ionic polymer (polysorbate 80) at the nanocapsule–water interface. The curcumin concentration in the nanocapsule suspensions was close to 1 mg/mL, corresponding to the intended theoretical concentration, which suggests that 100% encapsulation efficiency was achieved. These findings are consistent with previous studies reported by our group [29–31]. Acetone was removed by rotary evaporation under reduced pressure at 40 °C, a procedure widely employed in the nanoprecipitation-based preparation of polymeric nanocapsules. Considering the high volatility and water miscibility of acetone and its classification as a class-3 solvent (ICH Q3C), residual levels are expected to be minimal after evaporation. Nevertheless, residual solvent determination represents an important quality attribute for translation and regulatory purposes. Future studies will include validated headspace gas chromatography analysis in accordance with official guidelines to quantitatively assess residual solvents in the final formulation.

The three-layer 3D-printed films were rationally designed by combining hydrogels with complementary mechanical and biological functions to optimise skin adhesion, printability, and curcumin stability. The bottom layer, composed of chitosan, was selected to ensure direct contact with the skin, exploiting its cationic nature, which promotes electrostatic interactions with the negatively charged stratum corneum. This mechanism underlies its well-established bioadhesive behaviour, as well as its antimicrobial and wound-healing properties, which have been widely reported in the literature [32]. The intermediate layer consisted of a blend of Na-CMC and alginate, serving as the drug-loaded compartment. Na-CMC provides appropriate rheological properties for SSE, including shear-thinning behaviour and adequate yield stress, enabling accurate layer deposition and structural stability after printing, as previously demonstrated for SSE-based systems [27]. The incorporation of alginate contributes to biocompatibility and creates a hydrogel microenvironment resembling the extracellular matrix, which may favour tissue interaction [33]. The upper layer, composed of a CMC hydrogel containing titanium dioxide (TiO_2_), was designed to act as a photoprotective barrier. TiO_2_ acts as a physical UV filter by reflecting and scattering ultraviolet radiation, which is hypothesized as a strategy to reduce curcumin photodegradation [34–35]. Overall, this multilayer strategy highlights how SSE-based 3D printing enables the spatial separation of adhesion, drug delivery, and photoprotection functions, representing a significant advance over conventional single-layer topical films.

Regarding rheological properties, both yield stress and complex viscosity were estimated using an oscillatory amplitude sweep test. Yield stress is defined as the minimum stress required to initiate flow [36]. HG-CMC/Alg_c_ hydrogels required the highest stress to initiate flow, which is consistent with their higher complex viscosity values (Table 3). This behaviour can be attributed to the presence of ethanol, which was necessary for the solubilisation of curcumin in these formulations. Ethanol acts as a co-solvent and can significantly influence hydrogel rheology. Specifically, the reduction in the dielectric constant of the medium promotes intermolecular interactions between polymer chains, leading to a more compact and cohesive polymer network. This effect can result in increased yield stress and complex viscosity, particularly at moderate ethanol concentrations [37].

Results from the frequency oscillation test (see Figure 1D–F) are consistent with a predominantly elastic behaviour. Furthermore, all hydrogels (with or without curcumin-loaded nanocapsules) showed non-Newtonian flow behaviour, best fitting the Herschel–Bulkley model (*R*^2^ > 0.97), according to the equation τ = τ_0_ + *k*·*γ^n^* (τ = shear stress (Pa); τ_0_ = yield stress (Pa); *k* = consistency index (Pa·s^2^); γ = shear rate (1/s); *n* = flow behaviour index). This type of fluid requires a minimum applied stress to initiate flow (yield stress), which, in practical terms, corresponds to the force required to extrude the hydrogel through the syringe nozzle during printing. If the applied stress is below the yield stress, the hydrogel remains structurally intact inside the syringe. Based on the flow curves shown in Figure 1D–F, all hydrogels exhibited a decrease in apparent viscosity with increasing shear stress. This property, also known as shear-thinning behaviour, is particularly important for semi-solid materials used as printing inks, as it facilitates material extrusion while maintaining structural integrity after deposition [38–39].

Resulting from the high shear rates experienced within the printing nozzle, the cross-linked structure of the hydrogels may be affected during the 3D printing process. To monitor this effect, the thixotropic properties of the tested hydrogels were evaluated. This test involves measuring the viscosity of the hydrogels over three sequential shear intervals, known as the 3ITT, as a function of time, allowing the recovery of the initial viscosity to be assessed. An ideal hydrogel for SSE-based 3D printing should flow readily under applied stress and rapidly recover its rheological properties once the stress is removed. A recovery value of at least 80% is generally considered acceptable, indicating that the layer-stacking capability remains sufficiently dependent on the material’s original mechanical properties [40]. Initially, a shear strain of 1.0% was applied for 100 s to simulate the steady-state conditions within the syringe barrel prior to printing, followed by a shear strain of 100% for 100 s to reproduce the high-shear environment within the nozzle during extrusion. Finally, to simulate the post-printing condition, the initial shear conditions (1.0% for 100 s) were reapplied [41]. As shown in Table 4, all tested hydrogels achieved the minimum required recovery after stress cessation, indicating suitable thixotropic behaviour and confirming their appropriateness for 3D printing via the SSE technique.

The pH of the hydrogels was evaluated after dispersion of 1 g of hydrogel in 10 mL of ultrapure water. The results in Table S1 (Supporting Information File 1) show that all hydrogels have a slightly acidic pH, in the range of 4.5 to 6, making them suitable for skin applications [42]. Chitosan hydrogels showed the lowest pH values (4.7 ± 0.1 and 4.8 ± 0.1), due to the use of acetic acid during their preparation. As the nanocapsules were embedded within a continuous hydrogel matrix prior to printing, zeta potential was not measured after incorporation into the polymeric system. In this stage, nanoparticles are no longer dispersed in a liquid medium, and their stability is mainly governed by steric stabilization and physical confinement within the polymer network rather than electrostatic repulsion. The SSE process involves moderate shear stresses and short residence times, which are unlikely to induce irreversible nanoparticle aggregation. In addition, the hydrogel network acts as a steric barrier that limits nanoparticle mobility and reduces particle–particle contact during printing and drying. Previous studies have demonstrated the compatibility of SSE 3D printing with nanocarrier systems without significant loss of their nanometric characteristics [27].

After printing, films were left to dry at room temperature (25 °C) for 48 h, until a constant weight was achieved. The final weight of the 3D-printed films was 157.30 ± 4.58 mg for F_C-NC_ and 183.86 ± 6.55 mg for F_C_. Their drug content per film was 282.20 ± 7.75 µg in F_C-NC_ and 246.80 ± 6.70 µg in F_C_ films, corresponding to a recovery of (101.33 ± 0.22)% and (99.52 ± 0.45)%, respectively. For the 3D-printed films dispersed in water, pH measurements were 5.7 ± 0.2 for F_C-NC_ and 5.9 ± 0.7 for F_C_, in agreement with data previously discussed for the individual printing inks (hydrogels). These values are suitable for topical products, whose recommended pH values are between 4 and 6 [42].

DSC (Supporting Information File 1, Figure S1) analysis demonstrates that crystalline curcumin has an endothermic peak at 175 °C [43]. This signal was not observed in the physical mixture. In the F_C_ film, the absence of sharp endothermic transitions suggests molecular dispersion of curcumin within the polymeric matrix and the formation of intermolecular interactions, such as hydrogen bonding and hydrophobic associations, which likely contribute to reinforcement of the hydrogel network. In contrast, for F_C-NC_, the modified thermal event can be mainly attributed to the confinement of curcumin within the nanocarriers, which restricts molecular mobility and enhances the physical stability of the system. These structural differences are relevant to the performance of the printed material, as molecular dispersion in F_C_ may be associated with greater rheological rigidity and a faster initial drug release, whereas F_C-NC_ is expected to provide improved storage stability and controlled release profile [44–45].

The 3D films were intended to be used to facilitate skin repair, and wound healing treatment strongly depends on their bioadhesive properties, or more specifically, their skin adhesion properties. This property refers to bonding between components of a film and skin tissue surfaces, which can be achieved through both chemical and physical interactions. Lack of bioadhesion in such skin delivery formulations can limit their therapeutic efficacy and reduces clinical applicability, as they would require repeated applications [46]. In the present study, the influence of chitosan in the lower layer of the films was evaluated through a bioadhesion test, using a texture analyser and porcine ear skin.

Skin adhesiveness was measured as the force required to detach a porcine skin sample from specific films. Similar adhesive properties were measured for chitosan-containing films with either curcumin-loaded nanocapsules or unloaded curcumin (Figure 3A). The films prepared without a chitosan layer, containing unloaded curcumin, had lower skin adhesiveness than the analogous film containing a chitosan layer (*p* < 0.05). This result was expected because of the bioadhesive properties of chitosan [47]. Surprisingly, the same films, prepared without a chitosan layer, but with curcumin-loaded nanocapsules, did not show this difference, probably due to the inherent bioadhesive properties of the nanocarrier, as previously reported [48].

The work required to detach the skin sample from the 3D-printed films (bioadhesion work; Figure 3B) was also determined. Based on these measurements, the presence of nanocapsule carriers promoted higher skin adhesion in the films, in agreement with previous studies reported by Frank et al. [48], in which similar effects were discussed. These authors suggested that improvements in skin adhesiveness may be attributed to the high surface area of the nanocarriers, which allows for increased contact with the skin, while the polymeric composition of the nanocarriers may also contribute to enhanced adhesion. Taken together, these findings reinforce the interpretation presented above regarding the bioadhesive contribution of the nanocarriers used in the present study. Nevertheless, complete films containing a chitosan layer exhibited the highest bioadhesive capacity. Due to its cationic nature, chitosan is a well-known bioadhesive polymer capable of interacting with mucus glycoproteins [49–50]. Its positive charge enables strong interactions with negatively charged biological surfaces through electrostatic forces, while, under humid conditions, additional interactions with the stratum corneum may occur via hydrogen bonding and van der Waals forces [51].

Measurements of debonding distance (Figure 3C), which represents the distance travelled by the probe during complete displacement from the skin sample, agree with the bioadhesion findings discussed above. The longest debonding distance necessary to detach the probe from the skin sample was measured for films composed of all layers (including the bottom chitosan layer) and containing curcumin-loaded nanocapsules.

This proof-of-concept study focused on demonstrating the feasibility of integrating nanocarriers with SSE 3D printing to produce multilayer topical films with improved bioadhesion and photoprotection. A comprehensive evaluation of mechanical properties, long-term stability, and degradation behaviour was considered beyond the scope of this initial study and will be addressed in future formulation optimisation work. These attributes can be readily tuned in SSE 3D printing through adjustments in polymer composition, plasticizer content, layer thickness, and post-printing conditioning. Furthermore, morphological assessment was performed at both the nano- and the macroscale, using particle size distribution, polydispersity index, and zeta potential as indirect indicators of nanocapsule structural organization, while the morphology and integrity of the multilayer 3D-printed films were confirmed by visual inspection and dimensional analysis.

Finally, the effectiveness of TiO_2_ in the top layer for protection of curcumin from photodegradation was evaluated. The use of TiO_2_ in the rutile crystalline form was strategically selected due to its well-established ability to efficiently scatter and reflect ultraviolet radiation while exhibiting lower photocatalytic activity than the anatase form, thereby minimising the risk of ROS generation and contributing to the photostabilisation of curcumin within the multilayer film. Curcumin photodegradation has been associated with loss of biological activity and therapeutic efficacy [52]. Therefore, protection of curcumin from the effects of UV light exposure is an important step toward the development of successful therapies. Although UVC light is not encountered in natural sunlight, its use in this study was intentional as a stringent degradation condition to validate the protective function of the TiO_2_ top layer. As curcumin undergoes extremely rapid photolysis, UVC irradiation allowed for the discrimination between protected and unprotected films within a feasible analytical timeframe. Nevertheless, we acknowledge that evaluation under UVA/UVB illumination is required to ascertain real-world photostability, and these studies form part of our planned follow-up work. A mirrored chamber was used, and the concentration of curcumin in the 3D films was measured after exposure to a UVC lamp for 24 h. This evaluation was performed for films containing curcumin-loaded nanocapsules and unloaded curcumin, produced with or without the top layers composed of carboxymethylcellulose and TiO_2_. As shown in Figure 4, 24 h of UVC exposure resulted in decreased curcumin content in films produced without TiO_2_ in the top layer compared with those films containing a TiO_2_-containing top layer, whose curcumin content remained the same as at time *t* = 0. These findings support our initial hypothesis, demonstrating that the incorporation of a TiO_2_-containing top layer effectively protected curcumin from UVC-induced degradation, a result made possible by the multilayer design achievable only through the 3D printing process. Moreover, this effect was observed regardless of whether curcumin added to the film formulation was loaded into polymeric nanocarriers or not.

These findings highlight a key advantage of additive manufacturing, that is, the ability to rationally position functional components within distinct layers. In this system, bioadhesion, drug delivery, and photoprotection were structurally separated, yet integrated within a single construct. The data therefore support the central design hypothesis that the multilayer architecture, rather than nanocarrier incorporation alone, is the primary determinant of enhanced photostability.

A limitation of this study is the absence of in vitro release kinetics and skin permeation data, which are essential to fully characterise the biopharmaceutical performance of the films. These experiments were not required to test our central hypothesis that multilayer printing can provide enhanced bioadhesion and light protection. However, they will be crucial for future optimisation and translation, including in vitro release testing using Franz diffusion cells as well as skin permeation studies.

## Conclusion

In this proof-of-concept study, multilayer topical films containing curcumin were successfully fabricated by semi-solid extrusion (SSE) 3D printing, demonstrating the feasibility of additive manufacturing to design innovative structured polymeric systems for cutaneous delivery. The printing inks, formulated as hydrogels, exhibited non-Newtonian flow behaviour consistent with the Herschel–Bulkley model, confirming their suitability for SSE processing. The trilayer films were printed with reproducible curcumin content and an acidic pH compatible with topical application. Furthermore, the multilayer architecture enabled the functional evaluation of two design hypotheses, that is, first, that a chitosan-based bottom layer could enhance skin bioadhesion, and, second, that a TiO_2_-containing top layer could protect curcumin from light-induced degradation. Texture analysis confirmed that chitosan contributed measurably to the adhesive performance of the films, while photostability testing under UVC irradiation demonstrated that the TiO_2_ layer provided effective protection against curcumin degradation. Additionally, films containing nanoencapsulated curcumin showed increased bioadhesive performance relative to those containing free curcumin, supporting the contribution of nanocarriers to interfacial adhesion. Collectively, these findings validate the central hypothesis that SSE 3D printing can be used to rationally engineer multilayer polymeric films with distinct and complementary functions relevant to cutaneous delivery, including improved bioadhesion and enhanced photoprotection. The results demonstrate the potential of additive manufacturing to engineer hierarchically structured, nano-enabled systems with spatially resolved functionalities for advanced drug delivery. Further work is required to characterise drug release kinetics and skin permeation/penetration behaviour, which will be essential for advancing this printed multilayer system toward broader application in topical drug-delivery research.

## Supporting Information

File 1 Additional Figure and Table.

## Data Availability

Data generated and analyzed during this study is available from the corresponding author upon reasonable request.
