# Supplementary material for: Nanocarrier-integrated multilayer films produced by 3D printing for improved skin adhesion and curcumin photostability
Source: Beilstein J Nanotechnol. 2026 Mar 25;17:440–53. doi: 10.3762/bjnano.17.30 (PMC13040269; doi:10.3762/bjnano.17.30)
Supplement: File 1 — Additional Figure and Table. [file Beilstein_J_Nanotechnol-17-440-s001.pdf]

BEILSTEIN JOURNAL OF NANOTECHNOLOGY

Supporting Information

for

Nanocarrier-integrated multilayer films produced by 3D printing for improved skin adhesion and curcumin photostability

Thayse Viana de Oliveira, Ana Paula Farias Leão, Júlia Leão, Cesar Liberato Petzhold and Ruy Carlos Ruver Beck

Beilstein J. Nanotechnol. **2026,** *17,* 440–453. doi:10.3762/bjnano.17.30

Additional Figure and Table

License and Terms: This is a supporting information file under the terms of the Creative Commons Attribution License (https://creativecommons.org/licenses/by/4.0). Please note that the reuse, redistribution and reproduction in particular requires that the author(s) and source are credited and that individual graphics may be subject to special legal provisions.
The license is subject to the *Beilstein Journal of Nanotechnology* terms and conditions: (https://www.beilstein-journals.org/bjnano/terms)

Table S1: pH values for each hydrogel used in the 3D printing process (n = 3).[a]

Sample	pH
HG-CMC/TiO_2	5.30 ± 0.14
HG-CMC/Alg_{C-NC}	5.34 ± 0.18
HG-Chi_{C-NC}	4.76 ± 0.08
HG-CMC/Alg_{C-S}	5.85 ± 0.07
HG-Chi_{C-S}	4.67 ± 0.08

[a]C-NC: curcumin-loaded polymeric nanocapsules; HG-Chi_{C-NC}: chitosan hydrogel containing curcumin-loaded polymeric nanocapsules; HG-CMC/Alg_{C-NC}: blend of sodium carboxymethyl cellulose and alginate hydrogel containing curcumin-loaded polymeric nanocapsules; HG-Chi_{C-S}: chitosan hydrogel containing curcumin in hydroalcoholic solution; HG-CMC/Alg_{C-S}: blend of sodium carboxymethyl cellulose and alginate hydrogel containing curcumin in hydroalcoholic solution; HG-Chi: chitosan hydrogel; HG-CMC/Alg: blend of sodium carboxymethyl cellulose and alginate hydrogel; HG-CMC/TiO_2: blend of sodium carboxymethyl cellulose and alginate hydrogel containing titanium dioxide.

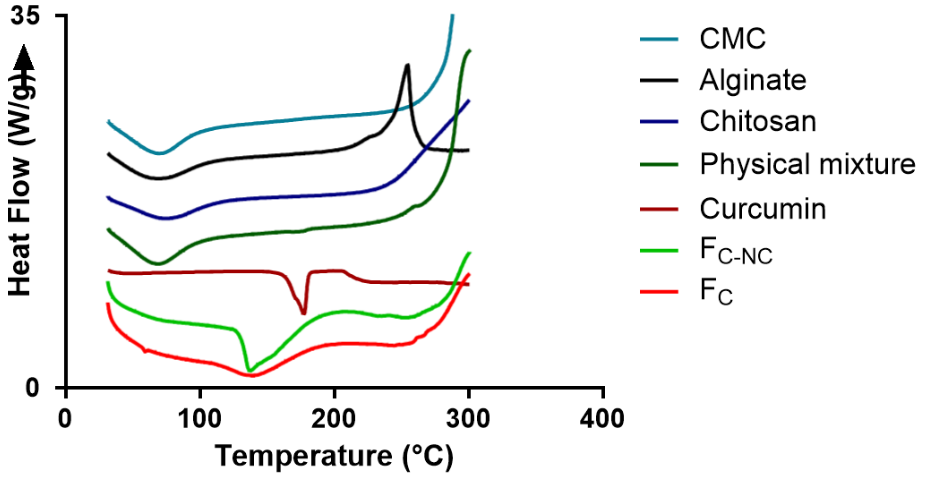

Figure S1: SM: Differential scanning calorimetry for curcumin and components of 3D-printed films. CMC: carboxymethyl cellulose, F_{C-NC}: 3D-printed film containing curcumin-loaded nanocapsules, and F_C: 3D-printed film containing unloaded curcumin.
